# Human germline genome editing is illegal in Canada, but could it be desirable for some members of the rare disease community?

**DOI:** 10.1007/s12687-019-00430-x

**Published:** 2019-08-16

**Authors:** Erika Kleiderman, Ian Norris Kellner Stedman

**Affiliations:** 1 Centre of Genomics and Policy, McGill University, 740, Dr. Penfield Avenue, suite 5200, Montreal, Quebec H3A 0G1 Canada; 2 Osgoode Hall Law School, 4700 Keele Street, Toronto, Ontario M3J 1P3 Canada

**Keywords:** Monogenic, Rare disease, Public health, Germline, Genome editing

## Abstract

Human germline genome editing may prove to be especially poignant for members of the rare disease community, many of whom are diagnosed with monogenic diseases. This community lacks broad representation in the literature surrounding genome editing, notably in Canada, yet is likely to be directly affected by eventual clinical applications of this technology. Although not generalizable, the literature does offer some commonalities regarding the experiences of rare disease patients. This manuscript seeks to contribute to the search for broader societal dialogue surrounding human germline genome editing by exploring some of those commonalities that comfort the notion that CRISPR may hold promise or be desirable for some members of this community. We first explore the legal and policy context surrounding germline genome editing, focusing closely on Canada, then provide an overview of the common challenges experienced by members of the rare disease community, and finally assess the opportunities of germline genome editing vis-à-vis rare disease as we advocate for the need to more actively engage with the community in our search for public engagement.

## Introduction

Human germline genome editing (HGGE) has piqued the scientific community’s interest over the past 3–4 years^1^ and is gaining traction in the public sphere. Experts who are deeply attentive to the technology’s possible uses disagree on whether it should be used in humans at all, but generally agree that CRISPR,^2^ an example of a genome-editing technique, has the potential to be transformative. When asked about what possible applications seem likely, many experts believe that CRISPR has great potential to cure and prevent serious genetic diseases. The most obvious category of disease is those caused by monogenic mutations in the human DNA (Lockyer 2016). This expected application may be especially poignant for members of the rare disease community, many of whom are diagnosed with monogenic diseases that come with high morbidity and mortality rates, and lack broad representation within the literature surrounding genome editing. While it is of course true that rare disease patients’ experiences are not generalizable, the literature on rare disease does offer some commonalities that may be instructive. We begin the manuscript by providing a brief overview of the Canadian legal and policy context surrounding HGGE, which provides an excellent starting point for a discussion about why broader societal dialogue should be encouraged. We then analyze the social science literature on rare diseases and look at the possibilities HGGE may hold for some members of this community. This manuscript seeks to contribute to the search for community engagement by arguing that commonalities found within the experiences of rare disease groups could add an important angle to the ongoing discussions and debates. Furthermore, these experiences could provide us with insight as to the potential desirability of HGGE for the treatment and prevention of serious or life-threatening genetic diseases^3^ and where the line should be drawn, if at all. We certainly do not wish to engage in a broad discussion about whether all rare disease patients share the same perspective. Rather, we wish to draw attention to the possibility that some members of the rare disease community, as a result of their many shared experiences, may perceive HGGE as particularly promising for themselves and their broader disease-specific communities.

## The law of genome editing in Canada

Genome editing is a technique used to make precise modifications to the DNA within a cell or an organism (e.g., bacteria, plants, animals, and humans). These modifications can be made to either somatic cells (i.e., limited to the individual) or to germ cells (i.e., heritable) (Ma et al. 2014). Applications of human genome editing can be for research purposes, therapeutic purposes, or non-therapeutic purposes (i.e., enhancement). In the context of this paper, we will focus on the human applications of HGGE and the potential opportunities that it offers for the prevention and treatment of disease, more specifically monogenic rare diseases.

In the midst of an active global dialogue about the merits and drawbacks of permitting the editing of human DNA in clinical settings, Canada is an example of a high-profile country that continues to have a criminal ban on HGGE (Isasi et al. 2016; Knoppers et al. 2017). Not alone in this position, a restrictive, statutory approach is most commonly used to address the concerns associated with HGGE. The 2004*Assisted Human Reproduction Act* (AHRA) governs human reproduction and related research and classifies HGGE as a criminal offense. The AHRA specifically states that “[n]o person shall knowingly […] alter the genome of a cell of a human being or *in vitro* embryo such that the alteration is capable of being transmitted to descendants” (s 5(1)(f)). Those who violate this provision may be guilty of an offense and subject to a fine up to $500,000 and/or 10 years in prison (s 60).

Canadian academics and clinicians engaging in the dialogue about whether to lift the criminal ban have an opportunity to inform the global debate despite Canadian clinician-researchers potentially falling behind their global colleagues based on the interpretive uncertainty surrounding the contextual application of the prohibition (i.e., research versus clinic) (Knoppers et al. 2017). Some researchers argue that this prohibition should only apply to reproductive uses of the technology, while allowing basic and pre-clinical research to move forward (Knoppers et al. 2017; Master and Bedford 2018). This would align with the Human Genome Editing Report that was released by the National Academies of Sciences, Engineering and Medicine, which stipulates that it may be permissible to move towards HGGE clinical trials one day, provided such trials be conducted for compelling reasons (e.g., prevention or treatment of serious or life-threatening diseases), meet a specific set of criteria, be subject to strict oversight, and be considered a “last” resort (i.e., no other reasonable alternatives exist) (National Academies of Sciences, Engineering, and Medicine 2017). Similarly, the Nuffield Council on Bioethics’ 2018 report entitled “Genome editing and human reproduction: social and ethical issues” states that HGGE is not unacceptable in itself; that is, it may become morally acceptable in time (Nuffield Council on Bioethics 2018). Recent statements on HGGE draw a clear distinction between research and clinical or reproductive purposes (de Lecuona et al. 2017; Federation of European Academies of Medicine (FEAM) 2017; National Academies of Sciences, Engineering, and Medicine 2017; Ormond et al. 2017; Brokowski 2018; De Wert et al. 2018). Therefore, we must not foreclose discussion so that we may engage with a variety of stakeholders to work towards a responsible solution (Baltimore et al. 2018; Nuffield Council on Bioethics 2018).

The momentum seems to be building in Canada for the criminal provisions in the AHRA to be reconsidered. There are other approaches to regulating research using HGGE that are less rigid than the current criminal prohibitions (Knoppers et al. 2017; Bubela et al. 2019; Knoppers and Kleiderman 2019). A principled approach could be adopted, for example, that is informed by evidence rather than driven by hype. This approach could allow policy to remain nuanced and as flexible as possible, when appropriate (Knoppers et al. 2017). Discussions about what this new regulatory framework would look like should be inclusive so as to have representation from various stakeholders, including the duly informed public (Knoppers et al. 2017; National Academies of Sciences, Engineering, and Medicine 2017; Rosenbaum 2019).

## Understanding rare disease

Rare disease patient organizations have been actively involved in the discussion about genome editing in Europe and the UK through organizations such as the European Organisation for Rare Diseases (European Parliament, the Council and the Commission 2012; EURORDIS 2017, 2018) and the Genetic Alliance UK; European Organisation for Rare Diseases (Genetic Alliance 2015; Genetic Alliance 2016; EURORDIS 2017; Luxner 2018). In Canada, on the other hand, the rare disease community has been rather quiet to date about genome editing. The definition of a rare disease varies by country, but it is typically based on the prevalence of a disease. For example, the *Orphan Drug Act* defines a rare disease as any condition that affects fewer than 200,000 people in the USA (Orphan Drug Act 1983, H.R. 5238 (97th)). Although very few people may have any given rare disease, many people will have a rare disease in their lifetime (Dodge et al. 2011). The Canadian Organization for Rare Disorders (CORD) estimates that rare diseases affect 1 in 12 Canadians and that approximately 7,000 rare diseases have been identified, many of which are genetic in origin. These numbers are growing as genetic testing becomes more ubiquitous in diagnostic medicine and as our understanding of disease becomes more precise.

Countries are also beginning to pay more attention to rare diseases within their healthcare systems (Dharssi et al. 2017). Unfortunately, the government of Canada has not yet adopted an official definition of what qualifies as a rare disease, nor has it implemented a comprehensive policy framework to attend to the needs of this community. Some political will existed in the past (CORD 2012), but it suffered a “kiss of death” in late 2017 when Health Canada removed its statement of intent to proceed with a regulatory framework from its website (Forrest 2017).

In part, conversations about HGGE reinforce a medical model of disability. The medical model considers disease as comprising categories of abnormality measured against the typical normalcy of a healthy human. For some, the prospect of allowing HGGE may represent a needless perpetuation of this medical model, whereas for others, HGGE may represent their only hope of having a healthy child (Polcz and Lewis 2016). Therefore, the goal of HGGE must be viewed, at least in part, as the fulfillment of parental desires of creating a healthy child through a medical intervention. We will return to this point later, after considering what the social model of disability can teach us about rare diseases.

The social model asks us to think of rare diseases as being more than mere differences from statistical norms, and to listen to the stories of individuals who experience rare diseases in order to locate those stories within the broader healthcare context (Newman 2014). In this context, disability and its associated harms are seen as the result of society’s organization (e.g., lack of available resources, stigma). The social model focuses on ways to further understand the barriers that limit life choices for disabled individuals so as to provide them with independence and control over their lives and options (Scope 2018). Sara Newman (2014) argues that the social model is a better approach for us to adopt in our efforts to understand rare diseases:

[…] advocacy groups use the social model to consider rare diseases within their broader contexts; by including the voices of those with rare diseases, and offering these materials online, these sites educate a wider public about the ways in which actual people experience these often diverse and complicated conditions (p. 53).

A third model that has been proposed by Savulescu and Kahane (2011) is the welfarist approach to disability. This approach incorporates aspects from both the medical and social models with a focus on the impact of a disease or condition on the well-being of the individual (Savulescu and Kahane 2011). As such, disease is perceived as relative to both the person and the circumstances (i.e., context matters) (Savulescu and Kahane 2011). If we wish to speak of rare diseases as a broad yet unified class of diseases, then the welfarist approach may offer a more inclusive lens through which to do this, than either the medical or social model taken independently.

Some, but not all, of the many challenges and experiences that connect rare disease patients can be found below in Table 1. Although this list has been compiled on the basis of a broad review of the literature, it is not exhaustive or comprehensive, and each item does not necessarily apply to every patient and/or family. It is also important to note that the way we have chosen to categorize and present the challenges and experiences within the table is simply to provide a general overview. These are not watertight compartments, and we recognize that the challenges and experiences presented in one category can, and often do, crossover into other categories.

**Table 1:** Connecting challenges and experiences for rare disease patients

Diagnosis	• Generally characterized as severe, chronic, progressive, and life-threatening (Esquivel-Sada and Nguyen 2018)—many rare diseases are associated with disability (de Chalendar et al. 2014); • More than half of rare diseases have an age of onset that occurs during childhood (CORD 2015); • Misdiagnosis is common and many patients experience the “diagnostic odyssey” (i.e., they may visit multiple doctors over multiple years to receive multiple wrong diagnoses) (Alonso et al. 2011; Baynam et al. 2016; Esquivel-Sada and Nguyen 2018); • It is not wholly uncommon for a patient to self-diagnose (Svenstrup et al. 2015; Goldman 2017); • Genetic testing can lead to incidental findings or variants of unknown significance (VUS)—which can complicate the diagnostic process (Kleiderman et al. 2014); • Patients can lose trust in the system and stop searching; • Diagnosis and information can assist families with reproductive decisions (Esquivel-Sada and Nguyen 2018); • Diagnosis can permit access to ancillary services, including social welfare, subsidies for special healthcare needs, access to support groups (Esquivel-Sada and Nguyen 2018).
Information/education	• Limited access to scientific and clinical experts due to a disease’s rarity; • Limited information about a condition(s), even after diagnosis; • Patients and families often become de facto experts in their disease and take on the burden of educating healthcare providers regarding standards of care; • Very little public awareness about most rare diseases.
Access	• Limited access to therapies or clinical trials (difficult to determine optimal study design for many diseases) (Mascalzoni et al. 2014; Kempf et al. 2018; Whicher et al. 2018); • Difficult to co-ordinate multi-center clinical trials because there are few patients and they can be difficult to locate^a^; • Expensive therapies may not be included on insurers’ formularies, making access challenging for those who are not wealthy. Legal recourse is relatively unavailable (Burningham 2015); • Therapies may be available in another jurisdiction, yet inaccessible due to restrictive laws and/or a patient’s limited ability to change where they reside (CTV News 2017; Marchitelli 2017; Ren and Labrie 2017).
Psycho-social	• Lonely and isolating (patients may never meet others with same diagnosis) (Huyard 2012); • Many parents may feel guilt from passing genetic disease to offspring; • Extreme stress from living with and/or caring for others (this can lead to family conflicts) (Esquivel-Sada and Nguyen 2018); • The experience of living with a rare disease or disability can change attitudes towards family planning and reproduction (Gollust et al. 2003); • Families dealing with disability frequently have an ambivalent relationship with reproductive genetics (Boardman and Hale 2018); • Asymmetry of services targeting pediatric and adult patients (Esquivel-Sada and Nguyen 2018); • Challenges with co-ordination of care because rare diseases can impact different systems within the body and often require a great deal of day-to-day support from various providers (pediatric and adult, at home and in facilities); • Perceived health-related quality of life is considered to be low (Bogart and Irvin 2017); • Social stigma can be brought on by physical appearance or other psychological and/or psycho-social challenges (Bogart and Irvin 2017); • Quality of life is often related to and impacted by the level of social stigma associated with a given disease or disability (Bogart and Irvin 2017).
Financial	• May require constant, time-consuming medical appointments; • Families suffer financially so they can care for the person/people impacted by the disease (Barrera and Galindo 2010).
Advocacy	• Disease becomes a significant part of many patients’ identity (can reshape what they see as their life goals) (Terry 2013); • There is a sense of community-building and positive identity forming for some (Panofsky 2011); • Many patients and families become unpaid advocates out of necessity—grassroots advocacy (Montini 2014); • Fundraising can be challenging because many small advocacy groups are competing with larger groups for limited charitable dollars from the public (Lordemann et al. 2014); • Advocacy groups tend to be funded by pharmaceutical companies interested in their disease – a practically unavoidable conflict of interest (Crowe 2018); • This conflict can cause public distrust in advocacy efforts.

^a^This could change in Ontario with the new Clinical Trials Ontario multi-center REB portal. See Clinical Trials Ontario. Streamlined Research Ethics Review System. http://www.ctontario.ca/streamlined-research-ethics-review-system/overview/. Accessed 26 February 2018.

## Germline genome editing through a rare disease lens

Roughly 7 million of the 130 million babies born worldwide each year have serious inherited genetic disorders (Church 2017). It is estimated that approximately 80% of those disorders are caused by a single gene mutation and “effective therapies for these diseases are themselves comparatively rare” (p. 681) (Boycott et al. 2013). The prevalence of rare monogenic disorders can vary depending on founder effect,^4^ geographic or cultural isolation, and genetic drift (Christianson et al. 2006), but the challenge of rare disease is global in its reach. It is therefore important to fully interrogate what the rare disease community’s perspective on germline editing might be.

Generally, somatic editing is seen as ethically acceptable, with several approved gene therapy clinical trials underway or expected to begin shortly (LePage 2017; Mullin 2017), whereas germline editing is more contentious. In August 2017, a highly anticipated study using CRISPR to correct a serious, heritable condition in human embryos made headlines. It was labeled a “landmark” study, being the first to successfully correct genes in viable human embryos with little to no off-target mutations or mosaicism (i.e., unexpected mutations or incomplete edits) which are some of the primary concerns surrounding germline editing, albeit in a research context (Ma et al. 2017). These findings are notable as they begin to address the relevant safety and efficacy issues related to germline editing and its potential future therapeutic applications. The possibility to repair mutations and prevent the reproductive transmission of heritable diseases is promising, especially for people with severe monogenic diseases that can cause significant morbidity or premature death. However, further research to confirm and continue to improve the safety and efficacy of the technology is still needed.

The reluctance to permit HGGE stems partly from the fact that preimplantation genetic diagnosis (PGD) is able to address many of the issues that might otherwise be addressed using this technology. PGD can reduce the risk of transmitting a heritable genetic mutation by screening and selecting against embryos that contain the mutation(s) in question. Although PGD is a valid option in the context of screening for healthy embryos, there are situations where PGD is simply not an option. For one, PGD cannot be used to make permanent changes to the germline that may edit out a hereditary genetic mutation. As such, PGD would not be a viable solution in rare cases where it can be expected that all embryos will necessarily carry a significant mutation, such as the case when one parent is homozygous for a dominant disorder (e.g., Huntington’s disease) or both parents are homozygous for a recessive disorder (e.g., cystic fibrosis) (Dance 2017; Hyun and Osborn 2017; Klipstein 2017).

Furthermore, people with rare monogenic disorders may never have access to effective therapies, proper care, or sufficient social support. The notion that PGD and somatic cell editing are sufficient solutions to the problem of monogenic rare disease seems to be driven by a medical model that concerns itself simply with eliminating disease while neglecting to consider impacts beyond the patient (i.e., the patient’s descendants). This approach pays inadequate attention to the social and cultural factors involved, the everyday challenges faced by rare disease patients and their families, and the implications of the disease on future generations. In this sense, PGD would remain the clinical standard, while HGGE could serve as an alternative approach that may enable couples to have a genetically related and healthy child when all other options fail (Cavaliere 2017).

Further concerns about HGGE arise due to its perceived negative ethical consequences. Some of the arguments raised in the literature are focused around human rights, including disability rights; social justice; intergenerational monitoring; human enhancement; and the need for greater public engagement. A brief outline of each of these will be presented below, along with possible responses that could be offered by the rare disease community.

To begin, we must address the disability rights literature. Originating from the expressivist objection to prenatal screening, advocates for people with disabilities (including people with rare diseases) have argued that prenatal screening and other selective reproductive technologies may cause further stigma (Asch 1999; Nelson 1998; Parens and Asch 2000; Wendell 2013). Selective reproductive technologies targeted at selecting against the birth of a disabled child perpetuate a certain hostility towards, and the discrimination faced by, individuals living with diseases and/or disabilities, as well as their families (Nelson 1998; Asch 1999; Parens and Asch 2000; Wendell 2013; Nuffield Council on Bioethics 2018). This same critique has also been leveled against the prospect of HGGE. It is argued that people with genetic diseases and/or disabilities “can have both high quality of life and value” (p. 1010) (Dance 2017), and eliminating particular diseases and/or disabilities suggests that “parents are unwilling to accept any significant departure from the parental dreams that a child’s characteristics might occasion” (p. S2) (Parens and Asch 1999). We must therefore consider the negative impact that eliminating genetic diseases and/or disabilities could have on the availability of social support and services for those who continue to need such resources (Dance 2017).

Some have also raised the importance of differentiating between disability and persons living with a disability, which puts into question the validity of the expressivist argument (Savulescu 2001). Essentially, the individual choice of parents (i.e., their reproductive freedom) to select or edit out a given disease in their future child should not be perceived as a general statement on or discrimination of people living with that disease (Savulescu 2001). Moving forward with HGGE could send a message that disability itself, and not societal discrimination against people with disabilities, is the problem to be solved. These disability rights arguments have been acknowledged in the HGGE literature and it is accepted that this tension between eliminating disease and respecting those who live with disabilities is one that we will likely never be able to fully resolve. Empirical work demonstrates that these concerns are real and that they do cause distress and offense to some people (Hofmann 2017; Nuffield Council on Bioethics 2018). That being said, the literature also suggests that “encouraging attempts to reduce the incidence of a genetic disease is compatible with continuing respect for those born with the disease and providing support for their distinctive needs” (p. 85) (Kitcher 1996). As such, it will continue to be important to engage in careful and open dialogue with stakeholders from the implicated disability communities about the social and moral implications of particular applications of the technology while we move towards the clinical use of genome editing.

The notion that the human genome represents the “heritage of humanity,” along with our general desire to prohibit activities that are contrary to human dignity, is enshrined in human rights principles (United Nations Educational and Scientific and Cultural Organization 1997). Under the *Oviedo Convention*, alterations to the human genome are permitted for “preventive, diagnostic, or therapeutic purposes” provided these changes are not made to the germline (Council of Europe 1997). As well, the notion of preventive health care is expressly included under the right to health found in article 35 of the *Charter of Fundamental Rights of the European Union* (European Parliament and the Council and the Commission 2012; Nordberg et al. 2017). Because these modifications are heritable and permanent, they can lead to unexpected health, epigenetic, and social consequences for future generations (Harris 2015; Dance 2017). As such, a human rights approach merits reflection in this context, as it can serve as a common starting point for the development of both national and international consensus regarding a responsible path forward for HGGE (Boggio et al. 2019; Knoppers and Kleiderman 2019). As Juengst (2017) argues, “[a] human rights approach to gene-editing policy would reorient our conversation from policing science to governing society and would shift our focus from avoiding risks to protecting opportunities” (p. 21).

We engage this same values discourse when we consider whether to eradicate diseases such as polio or measles. In the Western world, we have had the privilege of deciding that some diseases have no redeeming value and are worth eradicating because they can do incredible damage to the human race (So et al. 2017; Juengst et al. 2018). Many rare disease patients may argue that their diseases are more than mere differences, that they are solely harmful in nature, and that they have no redeeming value. Individuals born with Neonatal Onset Multisystem Inflammatory Disorder (NOMID), for example, must take an expensive immunosuppressant drug from birth if they wish to have a good chance of living into their teens. They must also be born in and continue to live in the right geographic location in order to have access to healthcare providers who understand their disease, can diagnose it, can prescribe and administer the necessary medication, and can conduct the ongoing testing needed in order to provide appropriate care. They would require sufficient health insurance to afford their medicine, there would need to be a specialty pharmacy where they live, and their insurance coverage would have to remain in place indefinitely. They may also have to reconsider their reproductive options/choice, factoring in the risk of bringing a child into this world who may experience the same challenges. Perhaps then, those very few people who live with a specific rare disease are in the best position to articulate what the eradication of their disease would signify to them. To date, the perspectives of various rare disease communities have not been sufficiently considered and responded to in the debates on HGGE. Yet, an emerging body of literature on the perspectives of people living with disabilities does exist and can be drawn on for guidance in the HGGE debate (Barnes 2014; Shakespeare 2015; Benjamin 2016; Saunders 2018). Such perspectives would undoubtedly add much depth to the conversation.

From a social justice standpoint, there is a concern that HGGE, if clinically available, would be expensive and limited to specialized centers. If so, then how would we determine who should have access and when? Should governments and insurers be required to guarantee equitable access (Dance 2017)? A rare disease patient might note that these inequities already exist and that they must live with them on a daily basis. In fact, healthcare inequities go much deeper than whether wealthy people are the only ones who have access to cutting-edge treatments. A person suffering from a monogenic rare disorder, for which there is no treatment, may already struggle daily with trying to grasp what justice the future holds for him or her and their family. It is reasonable to think that they would question the wisdom of banning HGGE on the basis of social justice concerns that ignore their own plight and the potential plight of their descendants.

A further concern is that current ethical guidelines for research involving humans do not account for the monitoring of individuals who are not part of the initial decision to participate in a study. Such monitoring would “expose future generations of the original participants’ families to potential abuse, exploitation, and psychological harms,” and raises concerns about the voluntary nature of their participation, protection of their privacy, re-identification, defining what “monitoring” would actually entail, and other unforeseeable risks (p. 1912) (Cwik 2017). Similar concerns arise with the introduction of any new or emerging medical technology, because a certain level of uncertainty is always present. If HGGE were eventually to become acceptable, it will be important to protect the welfare of children and future generations born from the technology, by ensuring proper ongoing follow-up and oversight, which in turn will require greater resources (Friedmann et al. 2015; National Academies of Sciences, Engineering, and Medicine 2017). There is little precedent in clinical research ethics when it comes to this notion of intergenerational monitoring (Cwik 2017). It involves not only the current participant (i.e., unborn child) but also their descendants (i.e., multiple generations), which is a unique consideration of HGGE; hence, such an approach will raise its own share of ethical and logistical issues that go beyond the scope of this manuscript. This monitoring not only includes the health of the individual but takes on a more holistic approach that necessitates consideration of the societal implications as well (Nuffield Council on Bioethics 2018).

The successful application of HGGE would have transgenerational benefits. Eradicating the single gene mutation from a family’s lineage reduces the risk of disease for their descendants (Gyngell et al. 2017). Concerns surrounding the difficulty of intergenerational monitoring arise, and they are reflective of a clinical research ethics system that needs to be updated in order to better account of the rise of genetics-based medicine (Cwik 2017; Hough and Ajetunmobi 2017; National Academies of Sciences, Engineering, and Medicine 2017). Despite the fact that the genetic causes for many rare diseases have been identified, most patients do not have effective therapies available to them and likely never will. Children born with many rare diseases languish in a system and suffer from delayed diagnosis, inconsistent access to proper medical care, and a lower than average quality of life, among many other challenges. Rare disease patients suffer disproportionately in our healthcare systems and HGGE could potentially relieve some needless suffering in the future. Therefore, to rare disease families, the challenges of intergenerational monitoring may be acceptable, provided that they allow for hope of a better quality of life for their descendants.

There is a general consensus that HGGE should not be used for enhancement purposes at this time (either cognitive or physical) (Brokowski 2018). Rather, research into therapeutic applications should be prioritized. Studies demonstrate that the public is supportive of using genome editing to cure or prevent life-threatening or debilitating diseases (including in embryos, children, and adults), but that germline enhancement is unacceptable (Blendon et al. 2016; McCaughey et al. 2016; Gaskell et al. 2017; Scheufele et al. 2017). The point of contention appears to be the application (i.e., therapy versus enhancement), rather than the technology itself. This distinction may not be particularly helpful, however, as the definition of “enhancement” seems to be a moving target (Dance 2017; Gaskell et al. 2017). We may be able to address this point of contention by adopting a blended governance model that includes ethics education, codes of professional conduct, regulation, government oversight, enforcement, and punishment, in order to minimize concern about the technology being used for needless human enhancement (Knoppers and Kleiderman 2019). Instead of looking to establish broad societal consensus about HGGE in general, we might look for what we call “group-specific consensus” that is driven by the needs and experiences of different groups. All the while, noting that there may not be consensus even among rare disease patients as experiences can differ quite dramatically. This approach would place our focus squarely on constructive applications targeting the elimination of serious diseases or conditions. Decisions about what constitutes a “serious” condition would be further contextualized and would give appropriate weight and consideration to the experiences and opinions of individuals who have lived with specific genetic diseases in embodied ways that few others will ever understand. Although we must recognize that many of the challenges posed by disease are social in nature, there is also a subset of the rare disease community for whom the medical model of disability is the most poignant. We must, therefore, engage with those for whom a reduction of the social barriers to health may be insufficient to address their condition. Accordingly, it may not be possible to give proper weight to subjective disease-specific experiences if we are intent on proceeding with HGGE only if we have “broad societal consensus.”

It is also important to note that a disconnect exists in the public’s understanding of the terms being used in the genome-editing debate, and public opinion is often skewed by media representations that either overhype or push dystopian views (Blendon et al. 2016). As such, mechanisms are needed to improve education, dispel misconceptions and misinformation, build trust, and increase public engagement in the issues surrounding HGGE (Nordberg et al. 2017; van Mil et al. 2017). It is also important to educate disease-specific groups about HGGE and the issues that arise, particularly within the rare disease community, so that they can contribute to the conversation about whether it is possible to achieve consensus about the structural and governance parameters that should be adopted in order to guide our use of this technology. This contribution will ultimately allow for better alignment of such parameters with the needs and concerns of the rare disease community.

Finally, there has been a call for groups who are seriously concerned with the applications of the technology to “organize politically to force policymakers to take the steps needed to prevent this technology from being abused” (Foht 2018). We wish to encourage disease-specific groups to take public positions about the prospect of HGGE so that their voices can be heard in these important ongoing public debates.

## Conclusion

Distinctive characteristics shared by members of rare disease communities make them uniquely positioned as a large group to take an active interest in the future of HGGE. Although some members of the rare disease community might object to HGGE for reasons that have been explored above, it is also clear that the burdens of rare disease can be multi-generational and devastating. Thus, some rare disease patients might challenge the consensus to oppose HGGE on the basis that this technology could provide them with hope of treatment, especially in cases where treatments or therapies are rare or nonexistent. The community is particularly well positioned to add a strong voice to the discussion that has yet to be heard. These discussions are timely and urgent, notably with the “CRISPR babies” scandal that shocked the international scientific and ethical communities in November 2018 (Kolata et al. 2018).

Even if the consensus is to not proceed with HGGE, the voice of the rare disease community can provide us with a great deal of insight as to the delineation of what may fall under the category of a serious disease worth eliminating. The real challenge in the discussion is sorting out whether and to what extent social conditions have a role in helping to define what qualifies as a “serious” disease—and whether the classification of “serious” is flexible depending on shifting social conditions and landscapes (e.g., if burdens are lessened through policies). There is a need to engage with the broader human community to help “guide and govern our technological futures” in a responsible and transparent manner, as decisions pertaining to HGGE belong to all of humanity (p. 135) (Hurlbut 2019).

## Footnotes

1 Genome editing was named method of the year in 2011, according to *Nature Methods*; however, it was not until 2014 that CRISPR-Cas9 really gained traction in international debates and was named breakthrough of the year in 2015, according to *Science*.

2 CRISPR: clustered regularly interspaced short palindromic repeats

3 For a deeper reflection on the notion of “serious” genetic disease, see: Wertz DC, Knoppers BM (2002) Serious genetic disorders: Can or should they be defined? American Journal of Medical Genetics 108:29–35 and Kleiderman E, Ravitsky V, & Knoppers BM (2019). The notion of “serious” and human germline genome modification. Journal of Medical Ethics [*submitted*].

4 The “founder effect” is typically defined as reduced genetic variation that accompanies the founding of a new (and often isolated) colony by a very small number of individuals of the original (larger) population. This effect can result in a new population that genetically differs considerably from the original population.

## References

[CR1] Alonso V, Villaverde-Hueso A, Hens M, Morales-Piga A, Abaitua I, de la Paz PM (2011). Public health research on rare diseases. Georgian Med News.

[CR2] Asch A (1999). Prenatal diagnosis and selective abortion: a challenge to practice and policy. Am J Public Health.

[CR3] Assisted Human Reproduction Act, SC 2004, c.2

[CR4] Baltimore D et al. (2018) Statement by the Organizing Committee of the Second International Summit on Human Genome Editing. http://www8.nationalacademies.org/onpinews/newsitem.aspx?RecordID=11282018b. Accessed 29 November 2018

[CR5] Barnes E (2014). Valuing disability, causing disability. Ethics.

[CR6] Barrera LA, Galindo GC (2010) Ethical aspects on rare diseases. In: Rare Diseases Epidemiology. Springer, pp 493–511 10.1007/978-90-481-9485-8_27 20824462

[CR7] Baynam G, Pachter N, McKenzie F, Townshend S, Slee J, Kiraly-Borri C, Vasudevan A, Hawkins A, Broley S, Schofield L, Verhoef H, Walker CE, Molster C, Blackwell JM, Jamieson S, Tang D, Lassmann T, Mina K, Beilby J, Davis M, Laing N, Murphy L, Weeramanthri T, Dawkins H, Goldblatt J (2016) The rare and undiagnosed diseases diagnostic service – application of massively parallel sequencing in a state-wide clinical service. Orphanet J Rare Dis:11, 77. 10.1186/s13023-016-0462-7 10.1186/s13023-016-0462-7 PMC4902909 27287197

[CR8] Benjamin R (2016). Interrogating equity: a disability justice approach to genetic engineering. Issues Sci Technol.

[CR9] Blendon RJ, Gorski MT, Benson JM (2016). The public and the gene-editing revolution. N Engl J Med.

[CR10] Boardman FK, Hale R (2018). How do genetically disabled adults view selective reproduction? Impairment, identity, and genetic screening. Mol Gen Genomic Med.

[CR11] Bogart KR, Irvin VL (2017). Health-related quality of life among adults with diverse rare disorders. Orphanet J Rare Dis.

[CR12] Boggio Andrea, Knoppers Bartha M., Almqvist Jessica, Romano Cesare P.R. (2019). The Human Right to Science and the Regulation of Human Germline Engineering. The CRISPR Journal.

[CR13] Boycott KM, Vanstone MR, Bulman DE, MacKenzie AE (2013). Rare-disease genetics in the era of next-generation sequencing: discovery to translation. Nat Rev Genet.

[CR14] Brokowski C (2018). Do CRISPR germline ethics statements cut it?. The CRISPR Journal.

[CR15] Bubela T, Kleiderman E, Master Z, Ogbogu U, Ravitsky V, Zarzeczny A, Knoppers BM (2019). Canada’s Assisted Human Reproduction Act: pragmatic reforms in support of research. Front Med.

[CR16] Burningham S (2015). Courts, challenges, and cures: legal avenues for patients with rare diseases to challenge health care decisions. Can J Comp Contemp Law.

[CR17] Canadian Organization for Rare Disorders (CORD) (2012) Canadian Organization for Rare Disorders welcomes announcement of orphan drug framework. https://www.newswire.ca/news-releases/canadian-organization-for-rare-disorders-welcomes-announcement-of-orphan-drug-framework-510864781.html. Accessed 6 March 2018

[CR18] Canadian Organization for Rare Disorders (CORD) (2015) Now is the time: a strategy for rare diseases in a strategy for all Canadians

[CR19] Cavaliere G (2017). Genome editing and assisted reproduction: curing embryos, society or prospective parents?. Med Health Care Philos.

[CR20] Christianson A, Howson CP, Modell B (2006). March of dimes: global report on birth defects: the hidden toll of dying and disabled children.

[CR21] Church G (2017). Compelling reasons for repairing human germlines. N Engl J Med.

[CR22] Council of Europe (1997) Convention for the protection of human rights and dignity of the human being with regard to the application of biology and medicine: convention on human rights and biomedicine (Oviedo Convention)

[CR23] Crowe K (2018) Following the money between patient groups and Big Pharma, *CBC News*. http://www.cbc.ca/news/health/second-opinion-patient-advocacy-pharmaceutical-industry-funding-drug-prices-1.4539271. Accessed 8 March 2018

[CR24] CTV News (2017) 63,000 Canadians left the country for medical treatment last year: Fraser Institute. https://www.ctvnews.ca/health/63-000-canadians-left-the-country-for-medical-treatment-last-year-fraser-institute-1.3486635. Accessed 6 March 2018

[CR25] Cwik B (2017). Designing ethical trials of germline gene editing. N Engl J Med.

[CR26] Dance A (2017). Better beings?. Nat Biotechnol.

[CR27] de Chalendar M, Daniel M, Olry A, Rath A (2014). Rare diseases and disabilities: improving the information available with three Orphanet projects. Orphanet J Rare Dis.

[CR28] de Lecuona I, Casado M, Marfany G, López-Baroni M, Escarrabill M (2017). Gene editing in humans: towards a global and inclusive debate for responsible research. Yale J Biol Med.

[CR29] De Wert G (2018). Human germline gene editing: recommendations of ESHG and ESHRE. Eur J Hum Genet.

[CR30] Dharssi S, Wong-Rieger D, Harold M, Terry S (2017). Review of 11 national policies for rare diseases in the context of key patient needs. Orphanet J Rare Dis.

[CR31] Dodge JA, Chigladze T, Donadieu J, Grossman Z, Ramos F, Serlicorni A, Siderius L, Stefanidis CJ, Tasic V, Valiulis A, Wierzba J (2011). The importance of rare diseases: from the gene to society. Arch Dis Child.

[CR32] Esquivel-Sada D, Nguyen MT (2018). Diagnosis of rare diseases under focus: impacts for Canadian patients. J Community Gen.

[CR33] European Organisation for Rare Diseases (EURORDIS) (2018)Introduction on the topic of genome editing for rare disease patients. http://download2.eurordis.org.s3.amazonaws.com/EURORDISIntroductionongenomeeditingforRDpatients.pdf. Accessed 25 July 2018

[CR34] European Organisation for Rare Diseases (EURORDIS) (2017) Rare disease patients & genome editing: perspectives and engagement. https://arrige.org/EURORDIS_CRISPR_meeting.pdf. Accessed 25 July 2018

[CR35] European Parliament, the Council and the Commission (2012) Charter of fundamental rights of the European Union. Official Journal of the European Union. 2012/C 326/02

[CR36] Federation of European Academies of Medicine (FEAM) (2017) The application of genome editing in humans: a position paper of FEAM - the Federation of European Academics of Medicine. https://www.feam.eu/wp-content/uploads/HumanGenomeEditingFEAMPositionPaper2017.pdf. Accessed 15 July 2018

[CR37] Foht B (2018) Gene editing: too much conversation, Not Enough Action. The Weekly Standard. https://www.weeklystandard.com/brendan-p-foht/gene-editing-too-much-conversation-not-enough-action. Accessed 25 June 2018

[CR38] Forrest M (2017) Health Canada gives ‘kiss of death’ to planned policy for rare-disease drugs. National Post. http://nationalpost.com/news/politics/health-canada-gives-kiss-of-death-to-planned-policy-for-rare-disease-drugs. Accessed 25 May 2018

[CR39] Friedmann T, Jonlin EC, King NMP, Torbett BE, Wivel NA, Kaneda Y, Sadelain M (2015). ASGCT and JSGT joint position statement on human genomic editing. Mol Ther.

[CR40] Gaskell G, Bard I, Allansdottir A, da Cunha RV, Eduard P, Hampel J, Hildt E, Hofmaier C, Kronberger N, Laursen S, Meijknecht A, Nordal S, Quintanilha A, Revuelta G, Saladié N, Sándor J, Santos JB, Seyringer S, Singh I, Somsen H, Toonders W, Torgersen H, Torre V, Varju M, Zwart H (2017). Public views on gene editing and its uses. Nat Biotechnol.

[CR41] Genetic Alliance UK (2015) Genome editing: what does it mean for patients? http://www.geneticalliance.org.uk/our-work/medical-research/genome-editing-what-does-it-mean-for-patients/. Accessed 25 July 2018

[CR42] Genetic Alliance UK (2016). Genome editing technologies: the patient perspective.

[CR43] Goldman B (2017) Man Googles rash, discovers he has one-in-a-million rare disease. *CBC Radio*. http://www.cbc.ca/radio/whitecoat/man-googles-rash-discovers-he-has-one-in-a-million-disease-1.4339872/man-googles-rash-discovers-he-has-one-in-a-million-rare-disease-1.4339923. Accessed 25 May 2018

[CR44] Gollust SE, Thompson RE, Gooding HC, Biesecker BB (2003). Living with achondroplasia: attitudes toward population screening and correlation with quality of life. Prenat Diagn.

[CR45] Gyngell C, Douglas T, Savulescu J (2017). The ethics of germline gene editing. J Appl Philos.

[CR46] Harris J (2015). Germline manipulation and our future worlds. Am J Bioeth.

[CR47] Hofmann B (2017). ‘You are inferior!’ Revisiting the expressivist argument. Bioethics.

[CR48] Hough SH, Ajetunmobi A (2017) The future of CRISPR applications in the lab, the clinic and society. In: Precision Medicine, CRISPR, and Genome Engineering. Springer, pp 157–178 10.1007/978-3-319-63904-8_9 29130159

[CR49] Hurlbut JB (2019). Human genome editing: ask whether, not how. Nature.

[CR50] Huyard C (2012). The emergence of the cause of rare diseases and rare disease patients’ movement. Orphanet J Rare Dis.

[CR51] Hyun I, Osborn C (2017). Query the merits of embryo editing for reproductive research now. Nat Biotechnol.

[CR52] Isasi R, Kleiderman E, Knoppers BM (2016). Editing policy to fit the genome?. Science.

[CR53] Juengst ET (2017). Crowdsourcing the moral limits of human gene editing?. Hast Cent Rep.

[CR54] Juengst ET, Henderson GE, Walker RL, Conley JM, MacKay D, Meagher KM, Saylor K, Waltz M, Kuczynski KJ, Cadigan RJ (2018). Is enhancement the price of prevention in human gene editing?. CRISPR J.

[CR55] Kempf L, Goldsmith JC, Temple R (2018). Challenges of developing and conducting clinical trials in rare disorders. Am J Med Genet A.

[CR56] Kitcher P (1996). The live to come: the genetic revolution and human possibilities.

[CR57] Kleiderman E, Knoppers BM, Fernandez CV, Boycott KM, Ouellette G, Wong-Rieger D, Adam S, Richer J, Avard D (2014). Returning incidental findings from genetic research to children: views of parents of children affected by rare diseases. J Med Ethics.

[CR58] Kleiderman E, Ravitsky V, Knoppers BM (2019) The "serious" factor in germline modification. Journal of Medical Ethics (in press) 10.1136/medethics-2019-105436 PMC6820154 31326898

[CR59] Klipstein Sigal (2017). Parenting in the Age of Preimplantation Gene Editing. Hastings Center Report.

[CR60] Knoppers BM (2017). Human gene editing: revisiting Canadian policy. Npj Regen Med.

[CR61] Knoppers BM, Kleiderman E (2019). “CRISPR babies”: what does this mean for science and Canada?. Can Med Assoc J.

[CR62] Kolata G, Wee S-L, Belluck P (2018) Chinese scientist claims to use CRISPR to make first genetically edited babies. https://www.nytimes.com/2018/11/26/health/gene-editing-babies-china.html. Accessed 13 May 2019

[CR63] LePage M (2017) Boom in human gene editing as 20 CRISPR trials gear up. New Scientist https://www.newscientist.com/article/2133095-boom-in-human-gene-editing-as-20-crispr-trials-gear-up/. Accessed 6 March 2018

[CR64] Lockyer EJ (2016). The potential of CRISPR-Cas9 for treating genetic disorders. Biosci Horizons: The International Journal of Student Research.

[CR65] Lordemann A, Danielsson K, Cheng-Ho Lin J, Bali RK, Bos L, Gibbons MC, Ibell S (2014). Innovative funding models for rare diseases. Rare Diseases in the Age of Health 2.0.

[CR66] Luxner L (2018) #ECRD2018 – genome editing might be ‘cure’ for rare diseases but ethical guidelines needed, panel says. ALS News Today. https://alsnewstoday.com/2018/05/30/ecrd2018-experts-debate-ethical-aspects-of-genome-editing-for-rare-disease-patients/. Accessed 25 July 2018

[CR67] Ma H, Marti-Gutierrez N, Park SW, Wu J, Lee Y, Suzuki K, Koski A, Ji D, Hayama T, Ahmed R, Darby H, van Dyken C, Li Y, Kang E, Park AR, Kim D, Kim ST, Gong J, Gu Y, Xu X, Battaglia D, Krieg SA, Lee DM, Wu DH, Wolf DP, Heitner SB, Belmonte JCI, Amato P, Kim JS, Kaul S, Mitalipov S (2017). Correction of a pathogenic gene mutation in human embryos. Nature.

[CR68] Ma Y, Zhang L, Huang X (2014). Genome modification by CRISPR/Cas9. FEBS J.

[CR69] Marchitelli R (2017) Location, location, location: how your health-care coverage is linked to where you live in Canada. *CBC News*. http://www.cbc.ca/news/canada/calgary/hearing-impaired-medical-implant-surgery-province-1.4435428. Accessed 6 March 2018

[CR70] Mascalzoni D, Paradiso A, Hansson M (2014). Rare disease research: breaking the privacy barrier. Appl Transl Genomics.

[CR71] Master Z, Bedford P (2018). CRISPR gene editing should be allowed in Canada, but under what circumstances?. J Obstet Gynaecol Can.

[CR72] McCaughey T, Sanfilippo PG, Gooden GEC, Budden DM, Fan L, Fenwick E, Rees G, MacGregor C, Si L, Chen C, Liang HH, Baldwin T, Pébay A, Hewitt AW (2016). A global social media survey of attitudes to human genome editing. Cell Stem Cell.

[CR73] Montini L, Bali RK, Bos L, Gibbons MC, Ibell S (2014). Health 2.0: the power of the internet to raise awareness of rare diseases. Rare diseases in the age of health 2.0.

[CR74] Mullin E (2017) CRISPR in 2018: coming to a human near you. MIT Technology Review. https://www.technologyreview.com/s/609722/crispr-in-2018-coming-to-a-human-near-you/. Accessed 6 March 2018

[CR75] National Academies of Sciences, Engineering, and Medicine (2017). Human genome editing: science, ethics, and governance.

[CR76] Nelson JL (1998). The meaning of the act: reflections on the expressive force of reproductive decision making and policies. Kennedy Instit Ethics J.

[CR77] Newman S, Rajeev KB, Bos L, Gibbons MC, Ibell S (2014). Rare diseases: the medical and the disability perspectives in the age of 2.0. Rare diseases in the age of health 2.0.

[CR78] Nordberg A, Minssen T, Holm S, Horst M, Mortensen K, Møller BL (2017). Cutting edges and weaving threads in the gene editing (Я) evolution: reconciling scientific progress with legal, ethical, and social concerns. J Law Biosci.

[CR79] Nuffield Council on Bioethics (2018). Genome editing and human reproduction: social and ethical issues.

[CR80] Ormond KE, Mortlock DP, Scholes DT, Bombard Y, Brody LC, Faucett WA, Garrison N’A, Hercher L, Isasi R, Middleton A, Musunuru K, Shriner D, Virani A, Young CE (2017). Human germline genome editing. Am J Hum Genet.

[CR81] Orphan Drug Act (1983) H.R. 5238 (97th)

[CR82] Panofsky A (2011). Generating sociability to drive science: patient advocacy organizations and genetics research. Soc Stud Sci.

[CR83] Parens E, Asch A (1999). The disability rights critique of prenatal genetic testing (special supplement). Hast Cent Rep.

[CR84] Parens E, Asch A (2000). Prenatal testing and disability rights.

[CR85] Polcz S, Lewis A (2016). CRISPR-Cas9 and the non-germline non-controversy. J Law Biosci.

[CR86] Ren F, Labrie Y (2017) Leaving Canada for medical care, 2017. Fraser Institute,

[CR87] Rosenbaum L (2019). The future of gene editing — toward scientific and social consensus. N Engl J Med.

[CR88] Saunders M (2018) Disability rights activists raise concerns over genetic editing for autism. https://theglobepost.com/2018/07/10/genetic-editing-autism-disability/. Accessed 29 May 2019

[CR89] Savulescu J (2001). Procreative beneficence: why we should select the best children. Bioethics.

[CR90] Savulescu J, Kahane G (2011). Disability: a welfarist approach. Clinical Ethics.

[CR91] Scheufele DA, Xenos MA, Howell EL, Rose KM, Brossard D, Hardy BW (2017). US attitudes on human genome editing. Science.

[CR92] Scope (2018) The social model of disability: what is it and why is it important? https://www.scope.org.uk/about-us/our-brand/social-model-of-disability. Accessed 6 March 2018

[CR93] Shakespeare T (2015). Gene editing: heed disability views. Nature.

[CR94] So D, Kleiderman E, Touré SB, Joly Y (2017). Disease resistance and the definition of genetic enhancement. Front Genet.

[CR95] Svenstrup D, Jørgensen HL, Winther O (2015). Rare disease diagnosis: a review of web search, social media and large-scale data-mining approaches. Rare Diseases.

[CR96] Terry S (2013). Disease advocacy organizations catalyze translational research. Front Genet.

[CR97] United Nations Educational, Scientific and Cultural Organization (UNESCO) (1997) Universal declaration on the human genome and human rights

[CR98] van Mil A, Hopkins H, Kinsella S, on behalf of the Royal Society (2017) Potential uses for genetic technologies: dialogue and engagement research conducted on behalf of the Royal Society

[CR99] Wendell S (2013). The rejected body: feminist philosophical reflections on disability.

[CR100] Wertz Dorothy C., Knoppers Bartha Maria (2002). Serious genetic disorders: Can or should they be defined?*. American Journal of Medical Genetics.

[CR101] Whicher D, Philbin S, Aronson N (2018). An overview of the impact of rare disease characteristics on research methodology. Orphanet J Rare Dis.

